# Community health workers and Covid-19: Cross-country evidence on their roles, experiences, challenges and adaptive strategies

**DOI:** 10.1371/journal.pgph.0001447

**Published:** 2023-01-04

**Authors:** Solomon Salve, Joanna Raven, Priya Das, Shuchi Srinivasan, Adiba Khaled, Mahwish Hayee, Gloria Olisenekwu, Kate Gooding

**Affiliations:** 1 Oxford Policy Management, Delhi, India; 2 Department of International Public Health, Liverpool School of Tropical Medicine, Liverpool, United Kingdom; 3 Oxford Policy Management, Dhaka, Bangladesh; 4 Oxford Policy Management, Islamabad, Pakistan; 5 Oxford Policy Management, Abuja, Nigeria; 6 Oxford Policy Management, Oxford, United Kingdom; York University, CANADA

## Abstract

Community health workers (CHWs) are a key part of the health workforce, with particular importance for reaching the most marginalised. CHWs’ contributions during pandemics have received growing attention, including for COVID-19. This paper contributes to learning about CHWs’ experiences during COVID-19, based on evidence from India, Bangladesh, Pakistan, Sierra Leone, Kenya and Ethiopia. The paper synthesises evidence from a set of research projects undertaken over 2020–2021. A thematic framework based on the research focus and related literature was used to code material from the reports. Following further analysis, interpretations were verified with the original research teams. CHWs made important contributions to the COVID-19 response, including in surveillance, community education, and support for people with COVID-19. There was some support for CHWs’ work, including training, personal protective equipment and financial incentives. However, support varied between countries, cadres and individual CHWs, and there were significant gaps, leaving CHWs vulnerable to infection and stress. CHWs also faced a range of other challenges, including health system issues such as disrupted medical supply chains, insufficient staff and high workloads, a particular difficulty for female CHWs who were balancing domestic responsibilities. Their work was also affected by COVID-19 public health measures, such as restrictions on gatherings and travel; and by supply-side constraints related to community access and attitudes, including distrust and stigmatization of CHWs as infectious or informers. CHWs demonstrated commitment in adapting their work, for example ensuring patients had adequate drugs in advance of lockdowns, and using their own money and time to address increased transport costs and higher workloads. Effectiveness of these adaptations varied, and some involved coping in a context of inadequate support. CHW are critical for effective response to disease outbreaks, including pandemics like COVID-19. To support CHWs’ contribution and protect their wellbeing, CHWs need adequate resources, managerial support, and motivation.

## Introduction

Community health workers (CHWs) are a key part of the health workforce in many low- and middle-income countries (LMICs), increasingly recognised as integral for effective and equitable health service delivery [1–4]. The umbrella term “CHW” encompasses diverse categories of health worker [4], such as community distributors, community-directed health workers, health auxiliaries, health promoters, family welfare educators, health volunteers, village health workers, community health aides, and barefoot doctors [5]. For this paper, we consider CHWs as health workers who are the first point of contact at community level, based in communities or at peripheral health posts, and who have some but fewer than two to three years of training [1,6]. Within this group, there is substantial diversity between countries and cadres in CHW responsibilities, skills and employment conditions [1,7].

CHWs undertake a wide range of tasks related to core health service provision, such as community mobilization, health promotion, and provision of preventive and clinical services [8,9]. Over the past decade, there has been a growing recognition of potential CHW roles in responding to pandemics [10]. Based in communities, and often from these same communities, CHWs are often the frontline and first point of contact when an outbreak hits [3]. For example, CHWs played multiple roles in the 2014 Ebola outbreak in West Africa [11–15]. Most recently, CHWs have been active in the COVID-19 response[16–23]. Research has documented the roles played by CHWs in controlling COVID-19, and the challenges they have experienced [16–24]. As well as introducing new roles for CHWs, COVID-19 has affected their routine service delivery. International surveys in 2020 and 2021 showed significant disruption to primary and community health services, with disruptions to community care reported by more than half the countries in the world in late 2021 [25–27]. In many countries, community services were deliberately suspended or scaled back, but services were also affected by issues such as disruption to supply chains, shortage of health workers and demand-side constraints [27].

This article contributes to the growing understanding of CHW roles and experiences in the COVID-19 response, synthesising evidence from a set of research projects in six countries in South Asia and Africa: Ethiopia, Kenya, Sierra Leone, Bangladesh, India and Pakistan. The article examines CHWs’ contributions to the response, the support provided to CHWs, and gaps in this support, challenges experienced by CHWs in delivering services, and adaptations or coping strategies that enabled service delivery. While guidelines were developed to support CHWs at an early stage in the COVID-19 pandemic [24], indications of widespread challenges for CHWs suggest a need for further evidence that can strengthen awareness and understanding of CHWs’ experiences and inform further measures to ensure they have adequate support.

The six countries included in this synthesis have all been significantly affected by COVID-19. All reported the first case during January–March 2020, and there was significant spread and community transmission by May [28,29]. COVID-19 added to other shocks experienced by these countries, including climatic shocks such as drought and floods in all 6 countries, and political instability or conflict in some, notably Ethiopia and Kenya. These countries are at different levels of development, but all face significant health system challenges–including financial constraints and health worker shortages, with all significantly below the WHO recommendation minimum of 44.5 skilled health workers (doctors, nurses and midwives) per 10,000 needed to achieve UHC and SDG3 [30] (see Table 1).

**Table 1:** Country development and health system status [31–33].

Country	Income classification	Human Development index	Skilled health workers per 10,000 population
Bangladesh	lower-middle income	133	6.02
Ethiopia	low-income	173	2.77
India	lower-middle income	131	27.49
Kenya	lower-middle income	143	10.66
Pakistan	lower-middle income	154	14.79
Sierra Leone	low-income	182	3.42

CHWs play a critical role in each country. Their specific titles vary between the countries (see Table 2 for information on cadres referred to in this paper). Some are voluntary positions, while others have more formal status and salaries, and their roles, levels of training and other employment conditions vary. In most countries, the majority of these CHWs are female.

**Table 2:** CHW cadres referred to in this paper.

Country	CHW Cadre	Abbreviation	Description (note that in some countries, roles and employment conditions vary between states/regions)
Bangladesh	Community Healthcare Provider	CHCP	Male and female government workers; three months of training; work full-time at community clinics; provide wide range of preventive, promotive, and curative care including managing childhood diarrhoea, pneumonia, and malaria; screening for malnutrition; new-born care, antenatal care (ANC) and postnatal care (PNC); and attending deliveries; receive monthly salary [34,35].
	Family Welfare Assistant	FWA	Female government worker; one month of training; work principally in rural areas at community and satellite clinics and through household visits; provide services related to family planning, ANC, PNC and child care, adolescent health, and nutrition; receive monthly salary [36,37].
	Family Welfare Visitor	FWV	Female government worker; 18 months’ training; work primarily in remote areas at union health and family welfare centres; provide family planning services, maternal and other services; receive monthly salary [38–40].
	Health Assistant	HA	Male and Female government worker; one month of training; work in community clinics and via household visits; focus on organizing immunization campaigns, including associated community education; receive monthly salary [34,37].
	Shasthya Shebikas	SS	Female non-government organization (NGO) volunteers, recruited by BRAC from village microfinance organisations; three weeks of training; work through household visits; provide basic treatment, health promotion and referral; receive performance-based incentives, and some income via selling medial products [34,37,41,42].
India	Auxiliary Nurse Midwife	ANM	Female health workers; eighteen months of training; based at health sub-centres (the lowest facility in the rural public health care system) and also conduct outreach; manage family planning, immunization, and maternal and child health programmes as well as other services; support Anganwadi Workers (AWWs) and Accredited Social Health Activists (ASHAs); receive monthly salary [43,44].
	Accredited Social Health Activist	ASHA	Female village-level voluntary health workers recruited by the local government village representatives; receive around 3 weeks’ initial training followed by further modules; mobilise communities to access health services, particularly for ANC, PNC and institutional delivery; receive performance-based incentives and/or a fixed salary, varying between states [45].
	Anganwadi Worker	AWW	Female nutrition and child development workers, recruited from the village; one month of training; run preschool Anganwadi Centres and provide early childhood health and education, provide nutritional supplementation for children, lactating and pregnant women, and adolescent girls, and support with other health services; receive a monthly honorarium [43].
	ASHA Facilitators	AF	Female health workers; trained in a two-day workshop; provide guidance, supervision and other support for ASHA, including practical support with service delivery such as community meetings, and data collection on key indicators related to ASHA work; receive monthly honorarium [44,46].
Kenya	Community Health Extension Worker	CHEW	Male and female government employees; trained for 6 months (classroom and field); provide health services at household and community levels, make referrals and linkages to health facilities, and provide supportive supervision to Community Health Volunteer (CHV); receive monthly salary [47,48].
	Community Health Volunteer	CHV	Male and female volunteers; three months training based on a curriculum with 13 modules,; deliver services in a defined geographical area location called a Community Health Unit (CHU); receive monthly incentives [37].
Pakistan	Lady Health Worker	LHW	Female government employees; three months of classroom training and additional on-the-job training; attached to a local health facility but primarily community-based, working from their homes and via household visits; initial focus on maternal and child health but now participate in large health campaigns, newborn care, community management of TB, and HIV/AIDS education; receive a monthly salary [49].
Sierra Leone	Community Health Worker	CHW	Male or female (approximately 24% female health workers employed by government; 24 days’ classroom training; work primarily via household visits and provide reproductive, maternal, new-born and child health services, health promotion and community mobilisation, and support surveillance; receive a monthly incentive and additional allowance for travel and other logistics [50,51].
Ethiopia	Health Extension Worker	HEWs	Primarily female (in most regions) government employees; one to two years’ training; work at health posts and through outreach and household visits; conduct health promotion, disease prevention, and basic treatment; receive a monthly salary [52].

## Methods

The paper synthesises evidence from a set of research projects conducted or supported by Oxford Policy Management (OPM) over 2020–21 (see Table 3 and further details in S1 Table). OPM is an international development consultancy organisation that works to support country governments and development partners through analysis and technical assistance across the policy cycle [53]. The projects that provided evidence for this synthesis were undertaken with a range of donor, research and country government partners, in India, Bangladesh, Pakistan, Sierra Leone, Kenya and Ethiopia. Projects and related reports were identified for inclusion based on author knowledge of OPM work and discussion with OPM country teams, with reports then reviewed to assess whether they provided relevant information.

**Table 3:** Projects and reports used for the synthesis.

Project	Title	Country	Focus	Methods
Assessing the Indirect Effects of COVID-19 on Essential Health and Nutrition Services in selected rural and urban settings of Bangladesh	Assessing the indirect effects of COVID-19 on Essential Health and Nutrition Services in selected rural and urban settings of Bangladesh: a qualitative study	Bangladesh	The indirect effects of COVID-19 on RMNCAH service provision, including coverage, quality and utilisation, including assessment of challenges and coping strategies used by health workers and communities	Document review and analysis of secondary data; interviews with government policy makers, development agencies, key experts, and with government, NGO, private and informal sector health service providers, including managers, CHW and other cadres; focus groups with CHW; focus groups and interviews with community members
Maintaining essential services after a natural disaster	COVID-19 Rapid country studies: country reports for Bangladesh, Kenya, and Sierra Leone, and cross-country synthesis covering these countries, Pakistan and Uganda	Bangladesh, Kenya, Pakistan, Sierra Leone, Uganda	Rapid situation analyses on the initial response to COVID-19 in the first few months of the outbreak, considering different health system pillars, wider governance structures, and response in related sectors such as social protection	Document review, key informant interviews with policy makers and other senior stakeholders
	Response and Preparedness for Essential Health and Nutrition Services During Disasters in Pakistan (including COVID-19)	Pakistan	Health system preparedness and response to shocks (floods, droughts, COVID-19), considering different health system pillars at national and province level, and community response	Interviews with national and district stakeholders, including government, NGOs, development agencies and other experts; health facility assessments using healthcare provider interviews; interviews with key community informants; focus group discussions with Lady Health Workers (LHWs) and community members; document review and review of HMIS data.
	The effectiveness of the Sierra Leone health sector response to health shocks: evidence from the COVID-19 perception survey	Sierra Leone	The health sector response to COVID-19, particularly related to service delivery, leadership and governance, the health workforce, and community ownership and participation.	Survey of Ministry of Health and Sanitation employees and stakeholders actively engaged with the COVID-19 outbreak response.
	Beyond the state: The role of traditional leaders in COVID-19	Sierra Leone	Role of traditional leaders in supporting the COVID-19 response at community level, including extent of and gaps in government coordination	Interviews with district government and traditional leaders
The Bihar Technical Support Programme	Bihar COVID-19: Experiences of Community Women (multiple reports)	India	Gender-based impacts of COVID-19 on the health, social lives, livelihoods and other aspects of women’s lives during COVID-19	Telephone interviews with female self-help group leaders, members and community mobilisers
	Bihar COVID-19 Situation: PHC Preparedness and Impact on Services (multiple reports).	India	Barriers and facilitators to the primary care response to COVID-19, the ability of rural health to provide routine essential services, and the vulnerabilities faced by health providers.	Document review; telephone conversations with PHC facility managers and frontline workers, including CHW
	Understanding the Community Health System: Experiences of Elected Women Representatives (EWRs)	India	The role of local government EWRs in the community health system, including their links with CHW, including their role during COVID-19	Telephone interviews with EWRs
	Making community health worker supervision more supportive: insights from an implementation research pilot.	India	The impact of training in supportive supervision alongside support for coaching and mentoring on the perceptions and performance of CHWs and their supervisors	Telephone interviews and in-person quantitative survey with CHW and supervisors
Assessment of Institutional Arrangements and Human Resources of the National Tuberculosis Elimination Program	Assessment of Institutional Arrangements and Human Resources of the National Tuberculosis Elimination Program (NTEP).	India	Institutional support mechanisms to implement the NTEP at national, state and district levels, with a focus on structures and gaps related to human resources, and including the impact of COVID-19 on TB services	Review of relevant policy documents; use of WHO Indicator of Staffing Need (WISN) to measure staff workload; Semi-structured telephone interviews.
Building Resilience in Ethiopia–Technical Assistance	Intra-Action Review (IAR) on Public Health Preparedness and Response to Covid-19 in Ethiopia: national report, and regional report for Gambella	Ethiopia	Effective practice and challenges in the COVID-19 response, to guide future action for the response and future preparedness	Document review; key informant interviews, focus group discussions and workshops with government staff involved in the response at national, regional, and facility level
Operational Research on Key Nutrition-Specific Intervention in Ethiopia	Formative research on key nutrition specific interventions in Ethiopia.	Ethiopia	Implementation of three nutrition-specific interventions, including gap and challenges, and the effects of COVID-19 on delivery	Interviews with health professionals at regional, zonal, health centre and health post levels, community interviews and focus groups, facility observation
Real time assessment (RTA) of UNICEF’s ongoing response to COVID-19 in eastern and southern Africa	Real time assessment (RTA) of UNICEF’s ongoing response to COVID-19 in eastern and southern Africa: COVID-19 vaccine supply and rollout.	Ethiopia, Rwanda, South Africa and South Sudan	Assessment of UNICEF’s support to COVID-19 vaccine rollout, including understanding progress and challenges in supply, distribution, and coordination	Document review, key informant interviews with staff from development partners and government at national and subnational levels

CHWs’ experiences during COVID-19 were the central focus for some projects, whereas others examined community health services, health system resilience, or the COVID-19 response broadly but included information on CHWs’ roles and experiences during COVID-19. Methods varied between the research projects, but were primarily qualitative, and included interviews, focus groups and document reviews (see Table 3). Some studies produced multiple reports, for example through monthly updates as the pandemic progressed. In total, 25 reports based on 18 studies were used for the synthesis. One report was publicly available (a real time assessment of UNICEF’s support to COVID-19 vaccine rollout [54], but the majority were internal for specific funders or other stakeholders.

To synthesise the material, a team of two researchers (SS and KG) conducted an initial rapid reading to map the range of information available. A thematic framework (see S2 Table) was then developed based on the research focus, knowledge of issues in the reports and other literature on CHWs’ roles and experiences with COVID-19. This framework was then adapted to clarify and add codes as reports were synthesised. Codes included roles that CHWs played during COVID-19, support provided, issues that helped or hindered CHW experience, effectiveness of service delivery, wellbeing of CHWs, and adaptation to enable continuation of service delivery. Taguette (an open-source tool for qualitative research available at https://www.taguette.org/) was used to import and code reports using the framework. Material under each theme was then exported for further analysis. Coding was led by one researcher (SS) and discussed among the research team (KG, JR) to support shared understanding of different themes as well as identification of new thematic areas.

Reports were not excluded on the basis of overall quality of research methods or analysis, as useful information can be obtained from reports that may otherwise have weaknesses [55]. However, reliability of report findings was considered for inclusion of specific information, for example, only drawing on reported findings with clearer underlying evidence, such as from empirical research where quotes or other evidence were sufficiently specific and detailed to be confident about interpretation. Most reports had been peer reviewed, both by senior OPM staff and external researchers, and all had some level of review from the organisations that commissioned the research.

Following initial drafting, synthesis findings were shared with a selection of researchers involved in conducting the original studies, to verify information and interpretation of their findings and so strengthen reliability of analysis. Discussion with these researchers also provided additional information from their research data or wider contextual understanding that was not included in the original project reports. This additional information was then incorporated in the analysis (where this additional information has been used in this paper, it is referenced as ‘project data’). Core OPM contacts for most projects are also authors for this article (SS, PD, GO, AK, KG). Including authors with different country contexts, professional affiliations and personal backgrounds helped to ensure that different interpretations and perspectives were considered in the analysis.

### Ethics statement

All the research studies included in the synthesis ensured that participants provided informed consent and that data was confidential. Consent was verbal or written, depending on the study approach (for example, remote interviews required verbal consent) and on context-specific understanding and guidance on appropriate procedures. Formal ethical review was provided for all reports (see S1 Table), except for two sets of rapid assessments conducted to inform government or other programming: the Intra-Action Reviews of the COVID-19 response in Ethiopia, and the rapid COVID-19 initial assessments conducted under the Maintains programme. These reports were focused on immediate feedback for programme decisions and restricted to document review and discussion with senior officials regarding health system issues; stakeholders and guidance indicated that approval was not required due to the low-risk approach and methods, restriction of interviews to areas of professional responsibility, and the focus on programme decisions.

## Results

In this section, we present findings from the different countries in line with the thematic framework developed for this synthesis, examining the roles played by CHWs in the COVID-19 response, the support they received–and gaps in this support, challenges that they experienced, and strategies used by CHWs to continue their work.

### What roles have CHWs played in the COVID-19 response?

In all countries, CHWs took on new roles in the COVID-19 response. Across countries, they contributed to aspects of case identification and surveillance, such as house screening, screening of people with COVID-19 symptoms, contact tracing, or reporting potential infections to district health teams [11,29,56–58]. Community engagement was another key role, including education and awareness on COVID-19 [57,59,60] and in India, counselling for returnee migrants [58]. In some countries, such as India and Bangladesh, CHWs were also involved in follow-up of COVID-19 patients including providing rations to those who were isolating and in need [59,61] and in Bangladesh, enforcing household lockdown for people with COVID-19 as well as returnee migrants [59]. Most of the research was conducted before COVID-19 vaccine rollout, so there was limited information on CHWs involvement in vaccine delivery. However, later reports indicated that CHWs in India and Ethiopia were involved in raising awareness about COVID-19 vaccination and encouraging uptake [54,62], and CHWs in Ethiopia were also involved in listing target populations and delivering vaccines [54]. CHWs’ responsibilities in delivery of routine essential services also changed during COVID-19. For example, in some areas of India, Accredited Social Health Activists (ASHAs) played new roles in both antenatal services (such as blood pressure checks), because the Auxiliary Nurse Midwife (ANMs) who would usually provide this antenatal care were unavailable, and in neonatal care (particularly first vaccinations), because of a rise in home deliveries [63,64].

As previously noted, the majority of CHWs in these countries are female. Reflecting existing gender-based occupational segregation in the health system, there were gender differences in assignment of COVID-19-related roles across the health workforce. In India, lower status positions are largely occupied by women, with very few women in management and leadership roles. Reflecting this, managerial, coordination and supervisory roles in COVID-19 were largely assigned to men, whereas female CHWs were primarily engaged in the more frontline activities such as screening and awareness raising [60].

### Support provided to enable CHWs’ work during COVID-19

CHWs received various forms of support for their roles in the COVID-19 response, as well as for conducting their routine service delivery during COVID-19. This included support through the formal health system, from the community, and through their personal relationships.

#### Support from the formal health system

Support through the formal health system included training and other guidance, personal protective equipment (PPE), financial support, and some support from managers. There were gaps in support in all these areas with consequences for CHWs’ ability to deliver services and for their well-being.

*Training*. Reports from most countries indicated provision of training for CHWs on services related to COVID-19, such as contact tracing and case management, and in some cases also on safe provision of routine services, including topics such as infection prevention and control and routine immunization during COVID-19 [29,65–67]. Development partners supported CHW training, including the United Nations Children’s Fund (UNICEF) in Sierra Leone and African Medical and Research Foundation (AMREF) Enterprises in Kenya [29,68].

To comply with physical distancing requirements and enable more rapid scale up of training, several countries conducted some training online or via mobile phones: in India, some training used zoom calls on mobile phones and the new ‘Disha’ App [67], Kenya used an application called ‘Leap’ to deliver learning content to CHWs by mobile phone [29], and Ethiopia relied on mobile-based and online training [69]. These approaches were reported to reach large numbers; for example, with 53,000 CHWs in Kenya reportedly trained via LEAP during early 2020 [29]. However, gaps in smartphone access meant these approaches could not reach all CHWs. In India, alongside use of online training, information and instructions on COVID-19 tasks were often sent by WhatsApp, but some ASHAs do not have any access to smartphones, and for many others, phones are kept by husbands [63,64]. There were also indications of wider gaps in training. For example, in India, some ANMs reported that they and Asha has not received training to support their work in raising awareness on COVID-19, and training had instead focused on management cadre. CHW often relied on public sources for information, such as television and WhatsApp messages [60].

*PPE*. CHWs across countries had insufficient PPE, with issues of quantity and quality. For example, some health assistants in Bangladesh received only three PPE sets over the first year of the pandemic, and shortages of masks and sanitiser were widely reported by ASHAs in India [59,70]. However, supply varied between CHWs and over time. For example, in Pakistan, some LHWs reported an absence of PPE while others reported both supplies and training on PPE use [56]. In India, initial shortages eased somewhat during later months (though with ongoing gaps for some ASHAs), and there were significant differences between cadres: ANMs in some cases received more PPE than ASHAs or AWWs [67], even though ASHAs had more contact with potential infection through their screening roles. ANMs also generally had stronger personal finances than ASHAs, and so could purchase PPE when it was not provided [63,64].

PPE shortages partly reflected systems constraints such as limited availability on international and national markets, and inadequate government budgets. However, shortages for CHWs also reflected issues of distribution. For example, in India and Kenya, available PPE was sometimes prioritised for those working on COVID-19 or in areas with a higher case load, leaving limited supplies for health workers providing routine services or in other locations [58,71]. In India, PPE distribution also reflected gendered health service hierarchies: guidelines on rational PPE use from the Ministry of Health and Family Welfare (MoHFW) categorised the (largely female) ASHAs as low-risk [58],and so lower priority for PPE, even though–as above—ASHAs were undertaking most screening and outreach. Some ANMs also reported that higher quality PPE (such as N.95 masks) was allocated to (often male) doctors and managers [67]. They voiced a sense of disempowerment and neglect, and described themselves during research discussions as “small people” (or lowest in the rung) for PPE distribution [67].

In the absence of PPE, some CHWs used inadequate substitutes. For example, in one state, some AWWs used hand towels, scarfs (*dupatta)* and handkerchiefs to cover their mouths when delivering home rations [58]. Some CHWs in Bangladesh and India purchased their own masks and hand sanitizer, sewed masks for themselves, or washed PPE for reuse [58,59].There was also some community support for availability of PPE: in India, some CHWs received masks distributed by local politicians, or made by local women’s self-help *mahila* groups [72].

Despite these efforts, limited and irregular supply of PPE left CHWs vulnerable to infection. This risk contributed to anxiety among CHWs, with fear both for their own safety, and of transmitting infection to their families [58,59]. For example, ASHAs and AWWs in India described fear of infection during household visits and community engagement due to insufficient PPE [58]. Broader fear of infection also led to reduced services elsewhere; for example, LHWs in Pakistan restricted their community outreach [56], and in Sierra Leone, some CHWs refused to come to work [73]. In addition, CHWs were also at risk due to patients either not having or not wearing PPE. For example, in Bangladesh, a healthcare assistant saw treating patients who did not wear masks as the biggest challenge faced in service delivery during COVID-19, prompting her to fear for her own safety and that of her family. Due to these concerns, some CHWs in Bangladesh chose not to serve patients who did not wear PPE [59].

When PPE was received, this enabled service delivery; for example, in Bangladesh, Shasthya Shebikas reported being able to resume TB sputum tests when they received personal safety equipment [59].

*Financial support*. In some countries, financial incentive schemes were introduced to recognise additional work or risks during COVID-19. In India, several incentives were announced by state or national government. For example, the central government announced in March 2020 that ASHAs would be paid INR (Indian rupees)1000 (13.10 USD) per month for three months for COVID-19-related activities, and provided insurance cover of INR 50 Lakhs (65,826 USD); and the government of one state announced remuneration of INR 400 (5.24 USD) for ASHA facilitators and 200 INR (2.62 USD) for ASHAs who undertook COVID-19 case finding surveys [58] Other announced support included 17,000 PKR (94.88 USD) per month for LHWs in Pakistan [56], and life insurance for health workers in Bangladesh [29].

Despite announcements, there were clear gaps in provision of incentives and information on this financial support. In India, in mid-2020, CHWs were often unaware of these entitlements, and additional incentives had often not been received [58]. They also saw compensation as limited in relation to the additional work required during COVID-19. Provision of incentives also varied between CHW cadres; for example, AWWs were not remunerated for their roles in COVID-19 in three states, while some ANMs and ASHAs in these states did receive additional allowances [58]. In some areas, AWWs went on strike due to lack of incentives for COVID-19, disrupting routine service delivery [74]. Elsewhere, there were examples of CHWs spending additional money out of their own pocket to provide services during COVID-19, without compensation; for example, HEWs in Ethiopia used their personal phone and air time to share information regarding COVID-19 with other health system levels [75].

Lack of financial compensation related to COVID-19 exacerbated the pre-existing situation of low and inconsistent pay. For example, in Ethiopia, HEWs reported demotivation due to low salaries, and in India, ASHAs are compensated through task-based incentives rather than a fixed salary in some states, and incentive and salary payments are often delayed, with additional disruption during COVID-19 [62].

#### Managerial support for CHWs during COVID-19

Given the challenges of work during COVID-19, support from supervisors had an important potential role in addressing new knowledge, safety and CHW well-being. There were examples of support from supervisors and managers; for example, in India, ASHA supervisors provided support during COVID-19 vaccination days [62]. However, COVID-19 often further reduced the quality of already inadequate supervision for ASHAs and AWWs: supervision became less frequent, was often conducted by phone (and so more difficult for ASHAs without easy phone access), and focused on disseminating instructions and discussing tasks related to COVID-19,rather than addressing CHWs’ concerns [76]. Inadequate advance planning and communication by higher level managers also created difficulties: some ASHA facilitators and ANMs reported only being told at 9PM about tasks for the following day, particularly related to COVID-19 vaccination. This hindered their ability to plan their work and arrange service delivery [62].

#### Support from peers, families and the community

Beyond support from the formal health system, some CHWs received support from peers, family and communities for their work during COVID-19. One area involves working additional hours to cover for colleagues who could not work; for example, in Bangladesh, some health assistants (HAs) worked additional days to cover for community health care provider (CHCP) who could not reach the clinic during lockdown transport restrictions [59]. However, as with formal health system support, there were mixed experiences. For example, in India, some CHWs in one state reported regular discussions among ASHAs, AWWs and ANMs as well as other stakeholders on COVID-19-related work such as management of returnee migrants and food distribution [58].However, many CHWs reported a lack of collaboration and support from their peers or other CHW cadres, with COVID-19 or routine activities [76].

Families were a source of support for some CHWs. For example, in India, when lockdown limited transport, some ANMs and ASHAs relied on their husbands and relatives to facilitate transport to facilities and communities [70].

There were also examples of support from community members. As well as the support with PPE discussed above, some CHWs reported support from communities with COVID-19-related work, for example with community members providing information, and self-help groups, community-based organisations and community volunteers providing support with activities related to quarantine, surveillance and awareness raising [70,77,78]. Elected Women’s Representative (EWRs), who work at ward level in one state of India, also provided support, for example by stepping in to conduct Anganwadi Worker (AWW service delivery activities when AWWs were on strike due to lack of incentives, or unable to travel due to lockdown measures [74].

### Challenges experienced by CHWs in providing services during COVID-19

CHWs experienced a range of challenges, both for activities related to the COVID-19 response as well as their routine service delivery. These challenges included issues related to the health system, such as limited human resources and gaps in supply chains; the effects of COVID-19 public health measures such as restrictions on gatherings and transport; and demand side issues related to community access and attitudes.

#### Health system factors

*Staff time and availability and increased workload*. Prevention and management activities related to COVID-19 added to CHWs’ existing workload, exacerbating already heavy workloads related to systemic health worker shortages [58,59,79]. In some cases, CHWs’ routine services were largely suspended, for example during the 2020 lockdown in India, but where routine services were expected to continue, this was difficult for CHWs to manage alongside new COVID-19 responsibilities. For example, in Ethiopia, new tasks such as community awareness on COVID-19, led HEW to deprioritise their nutrition services [79]. In Bangladesh, additional activities were transferred from colleagues who could not work, for example due to being classified as vulnerable, or because they were in isolation as COVID-19 patients. Lack of staff in community clinics meant health assistants (HAs) worked overtime each day and at weekends to manage the patient load and to conduct home visits to COVID-19 patients [59].

In India, in some states, some ANMs worked in facilities (to ease the shortage of general nurses), while some focused on outreach, and others played a dual outreach and facility role. During the initial COVID-19 stages, outreach ANMs were reoriented to work in facilities and quarantine centres. When their outreach activities resumed, they were stretched between COVID-19-related work such as surveys, screening and quarantine management, and conducting their routine immunisation and ANC services. In addition, without support from outreach ANMs and with resumption of routine activities, facility ANMs struggled to provide services across cold chain management, the labour room, emergency care and outpatient department, particularly as COVID-19 screening and registration of returnee migrants increased facility workloads [58,67]. COVID-19 also brought long hours, with all CHW leave cancelled, and notable gender variations: due to their role in screening and quarantine, ANMs were instructed to be available 24/7, and go to the field at night if needed, something not requested for male facility staff [60].

Where workload and hours increased, this created stress for CHWs, particularly with trying to balance additional work with domestic responsibilities. For example, ASHAs in one state described stress related to balancing household chores with additional COVID-19 work, such as contact tracing, and uncertain hours [76]. Afs in one state also faced difficulty in managing additional workloads alongside domestic responsibilities, particularly as the household work burden increased with return of some family members during COVID-19 [60]. However, there were also examples of ANMs who lived on their own and so had fewer household responsibilities, who were able, or required, to work additional hours, including night shifts, to cover for ANMs with more family duties.

*Medical supplies*. Across countries, disruption to medical supply chains led to shortages, often exacerbating existing difficulties [11,29,58,65,79]. This affected the supplies needed by CHWs for their work. For example, in Ethiopia, COVID-19 affected supply of iron and folic acid and vitamin A distributed by HEWs [79]. In Bangladesh, COVID-19 lockdown restrictions hindered transport of medical supplies needed by CHWs. Transport difficulties also increased use of community clinics as people could not access higher level facilities, which led to a shortage of medicine at community level [59]. In India, lockdown disrupted supply of the rations distributed by AWWs [72], and some ASHAs reported shortage of supplies such as paracetamol, oral rehydration sachets and contraceptives; supplies were inconsistent pre-COVID-19, but shortages were exacerbated by increased supply chain disruption [72]. The situation improved in later months, with resumed availability of supplies and rations [72].

#### Restrictions related to COVID-19 public health measures

Measures taken to prevent and control COVID-19 also affected CHWs’ service delivery. In particular, national guidelines and regulations on gathering and physical distancing affected whether services were allowed to operate or seen as possible, and wider movement restrictions and associated impacts on travel affected CHWs’ ability to access their place of work and communities.

CHWs’ home visits and community outreach activities were suspended during initial periods in several countries, with some routine Reproductive, Maternal, Neonatal and Child Health services not functioning during lockdowns [56,58,59,79]. For example, in India, community outreach services days for ANC and immunisation and the Village Health Sanitation and Nutrition Days were paused partly due to guidelines against group gatherings [60]. In Ethiopia, COVID-19 precautions around physical distancing made it more difficult for HEWs to provide services such as nutrition screening, which are usually conducted via community gatherings [79]. In Bangladesh, several CHW services were suspended, such as immunisation, satellite clinics and household visits by Family Welfare Assistants (FWAs) for activities such as distributing contraceptives. Follow up visits to people with tuberculosis were also suspended [59]. Physical distancing requirements also limited CHWs’ work when services were provided. For example, in Bangladesh, CHWs discussed the limiting effect of safety distance on ability to conduct examinations for ANC or to check blood pressure for patients with diabetes [59].

Movement restrictions and disruption to transport related to lockdowns also hindered CHWs’ service delivery, limiting their ability to visit health facilities and communities [11,58,59,72,76]. In Bangladesh, limitations on transport and movement made CCHPs’ daily commute to the community clinic more difficult, and meant some HAs had to walk to collect COVID-19 samples from patients rather than using transport. They also hindered ability of BRAC Shasthya Shebikas to meet TB patients, affecting continuity of care, and FWAs were unable to provide household services or set up Satellite Clinics. Increased transport fares also brought additional expenses for CHWs, in some cases leading them to reduce spending on other family essentials to cover transport costs [59]. In India, transport shortages made it hard for AWWs to collect and distribute home rations [58], and combined with reallocation of ambulances to the COVID-19 response, transport shortages made it difficult for ASHAs to conduct their role of escorting women for institutional deliveries [70], and in some cases meant ASHAs used their own money to pay for taxi services. Travel restrictions also made it difficult for HEWs to visit communities in Ethiopia [79].

#### Community access, demand and beliefs

Travel restrictions also affected the demand side of health services by reducing community ability to access care [79,80]. For example, in India, ANC services provided at community health centres were often paused when these facilities were converted to quarantine centres, but even when these centres or private facilities were providing services, lack of transport hindered women’s ability to access them for ANC [80].

Community fears of infection or quarantine also affected CHWs’ services. For example, in Ethiopia and India, fear of infection reduced community visits to facilities [79,80]. In Bangladesh and India, some community members did not allow CHWs to enter their homes for visits due to concern about infection [59,81]. Community concerns about stigma and fear of quarantine due to being identified as having COVID-19 also reduced community demand, and affected community trust in CHWs and CHWs’ ability to perform their tasks. For example, in India, CHWs were expected to identify people with potential COVID-19, but community members were often reluctant to report symptoms due to fear of stigma or quarantine [72]. CHWs were also tasked with monitoring returnees, but migrant families did not want to provide phone numbers nor allow CHWs to paste quarantine slips used to indicate families potentially affected by COVID-19 on their doors [76,78,81].Broader political contexts also contributed to concern and reluctance, for example with wider tensions between religious groups and misperception of particular groups as increasing the COVID-19 spread contributing to fear of COVID-19 screening among some sections of the community [67].

Community concerns about infection, quarantine and stigma also led to stigmatization of CHWs and in some cases, aggression from community members. For example, in Sierra Leone, some CHWs experienced stigmatisation as carriers of COVID-19 [11]. In Bangladesh, CHWs were refused access to public transport when wearing PPE, presumably because this indicated that they had been in contact with people potentially affected by COVID-19 [59]. In India, there were examples of aggressive behaviour towards ASHAs from the families of people identified as having COVID-19, and suspicion that CHWs were working as informers to facilities or the police [58].

#### Adaptations to enable service delivery

Several approaches were adopted by CHWs to adapt and continue work during COVID-19, partly through their own initiative but also directed by more senior health system managers and guidelines. The use of telemedicine was widespread, helping to reach clients when face to face contact was not possible due to movement restrictions or client concerns [56,59,67,79]. In several countries, CHWs used phone calls to provide maternal health services. For example, in India, ANMs used phone calls to pregnant women to check their health and screen for COVID-19 symptoms [70], and ASHAs phoned pregnant women to provide guidance and counselling on nutrition and precautionary measures during COVID-19 when facilities were closed [77]. Similarly in Pakistan, LHWs in parts of Karachi used phone calls to remain in contact with pregnant women and postnatal women when households were reluctant to have LHWs visit [56]. In Bangladesh, FWVs and CHCPs provided counselling via phone when lockdowns prevented community visits. BRAC Shasthya Shebikas also used phone calls to follow up TB patients for compliance with treatment [59]. While this enabled some service continuity, some CHWs in Bangladesh felt it reduced quality of service provision and hindered interpersonal communication [59]. In addition, low access to phones by community members meant these approaches could not reach all those in need, as seen in India with use of phones by AWWs, ASHAs and ANMs to communicate with community members [63,64].

Another set of adaptations is related to provision of medical supplies. In Bangladesh, knowing that the coming March 2020 lockdown would prevent household visits, FWAs provided clients with additional quantities of oral pills and male condoms in advance. CHWs also advised on alternative contraceptives when lockdowns prevented injections [59]. TB patients were also provided with additional medicines in Bangladesh [59] and India [82]. However, there were also examples of CHWs reducing provision of drugs to avoid stockouts: in Bangladesh, some CHCPs rationed the supply of medicines for patients to delay stockouts [59]. There were also adaptations to ensure CHWs had access to drugs when movement restrictions disrupted supply chains. In Bangladesh, when road barricades were imposed to restrict travel, BRAC sent TB medicines up to barricades, from where Shasthya Shebikas then collected them. For remote villages, the medicines would then be relayed through multiple Shasthya Shebikas [59].

There were also changes in service delivery locations. For example, in Bangladesh, when immunisation sessions could not be held in people’s homes, CHWs instead used schools, as they were closed and so available, and provided more space [59]. In Pakistan, LHWs provided maternity services for pregnant women from their own homes [56], and in Bangladesh, some CHWs supplied TB medications or contraceptives from their own homes when facilities were closed or CHWs could not make household visits [59]. In India, when community health days were stopped, ASHAs instead provided routine services such as nutrition via household visits, and AWWs provided rations via household visits (or collection from the AWW’s house) because anganwadi centres were closed [58]. Other adaptations included changes to the structure of service delivery. For example, in Ethiopia, some services delivered by HEWs were provided as campaigns to catch up on missed appointments, some HEWs divided villages into smaller groups to enable services despite restrictions on large gatherings [79]. Other examples include making use of COVID-19-related activities to provide other services: in India, there were examples of some district officials advising ASHAs to use household visits for COVID-19 surveys to also provide routine services such as those related to ANC, child health and TB [82].

Beyond the strategies above, some CHWs found additional ways to maintain services and support communities. These strategies often demonstrated personal commitment, including long working hours (as shown in 4.3.1). For example, ASHAs in India were busy with COVID-19 response activities during the day and so worked at night to escort pregnant women to facilities for delivery [70]. CHWs also used their own financial resources to overcome constraints, including purchasing their own PPE and covering extra transport costs as indicated in section 4.3. There were also examples of CHWs providing support to community members in need; for example, some LHWs in Pakistan distributed rations on their own when rising food prices and falls in income due to lockdowns left household vulnerable to malnutrition [56]. However, while demonstrating commitment, adaptations were also associated with a lack of choice and power, with many CHWs in India describing their situation during COVID-19 as involving ’majboori’, or helplessness and lack of options [64,67].

## Discussion

This paper contributes to growing learning about CHW roles. We know that CHWs are a critical component of the workforce providing health care services to communities and often reach the most marginalised groups, and that they need support to be able to fulfil this vital role [1]. Our findings highlight these issues in the context of COVID-19: they show CHW contributions to pandemic response; the influence of health system factors, wider government decisions, gendered hierarchies and community relationships on their work; the effects of inadequate support and other challenges on CHWs’ wellbeing; and CHWs’ agency and commitment in continuing service delivery; and variation in experience between CHW. The findings can inform strategies to support CHW roles and wellbeing during shocks such as pandemics, so contributing to strengthening health system resilience in shock-prone settings.

### The ever-expanding role of the CHW

CHWs continued to play crucial roles in providing routine services in all countries in our study. Our synthesis showed that apart from routine service delivery CHWs were actively involved in COVID-19 activities such as contact tracing, community education and awareness on COVID-19, screening people with potential COVID-19 symptoms, counselling migrants. Similar roles in both the COVID-19 response and continued delivery of routine services during COVID-19 have been identified by other research, both in some countries included in our research, such as India, Sierra Leone and Kenya and in other contexts, as well as in global syntheses [8,16,18,22,83–85].

The potential for CHWs to support delivery of COVID-19 vaccines has also been recognised [86,87]. CHWs on the whole enjoy trust and are often embedded within their communities which enables them to take on a role in vaccine delivery [51]. Due to the relatively recent timing of COVID-19 vaccine rollout, there was limited information on CHWs’ roles in vaccination, both in the research used for this article and in the wider literature, but our paper indicates examples of CHWs contributions to vaccine uptake, and to some extent delivery.

However, there are challenges with this ever-expanding role of the CHW. They are often the funnel through which all programmes are delivered at the community level, requiring them to acquire new skills and knowledge, and cope with an increasing workload. Understanding the challenges CHWs face is critical to providing support that enables them to take on and sustain these roles.

### CHWs need a package of support

Our synthesis shows that some support from the health system, peers, community and family was provided to CHWs as they took on new responsibilities and continued with their existing tasks. However, there were significant gaps in this support.

Health systems in all countries were ill-prepared to face the challenges of the COVID-19 pandemic [88–91]. This is reflected in the global level shortages of PPE [92], which resulted in inadequate supply to CHWs. This has implications on CHW’s ability and willingness to deliver services, and increased fear of infection for themselves as well as their families. Allocating PPE to the health workforce must consider the critical role of CHWs in pandemic response [23].

For CHWs to effectively take on new roles, training and supervision is essential. Our synthesis shows that although some training was provided, there were gaps. Supervision was also largely absent. Timely, quality training, and refresher training, are needed, and supervision is equally important, especially when responsibilities are new, complex, often stressful, and require sensitivity. Telephone, peer and group supervision that includes space to reflect and problem solve on CHWs’ experiences could help to reduce over stretched facility level supervisors [51].

From our synthesis, plans to provide additional financial incentives to CHWs working in COVID-19 did not always materialise. In some settings, CHWs were unaware that they should receive these. This finding is consistent with other studies on financial incentives for CHWs, and particularly in fragile settings, where livelihoods are often precarious, CHWs rely on the financial incentives provided for their role [93,94]. Studies show financial performance-based incentives can improve CHWs service delivery outcomes [95], and needs consideration in pandemic situations as well.

Our synthesis has shown that some CHWs experience mental distress and anxiety, as well as discrimination and stigma from family and community members. Similar issues have been identified in other studies during COVID-19 or other epidemics [17,96–98]. CHWs’ mental health and well-being needs to be protected. Recognition of the effects of the pandemic on mental health of CHWs is the first step, but this needs to be followed with provision of ongoing support for mental health and coping mechanisms, from health system actors and peers, families, and communities [96], during and after the COVID-19 pandemic [17,97]. We found that peer support can help with coping with the additional stress of the COVID-19 pandemic, as also highlighted in another recent review [98]. Photovoice method, albeit used more often in research, can also create space for CHWs to not only support each other but also jointly problem solve [51].

We also identified that families and community members can be a source of support, and can help CHWs carry out their responsibilities as seen in other studies [99]. However, there were also instances showing that family and community members can ostracise CHW because of fear of infection or misconceptions about COVID-19 or CHWs’ roles. This shows the need for clear messaging on CHWs’ roles and strategies to address stigma.

Many CHWs experience difficult working conditions and weak employment status during routine times [100], as noted in our synthesis in relation to the normality of fragile financial payments. Action taken to support CHWs during COVID-19 should address the systemic issues affecting CHWs, going beyond short-term measures to support stronger ongoing health system resilience and CHWs well-being [83,100].

### CHWs show resilience and adapt their way of working

Despite numerous challenges, CHWs showed individual resilience, adapting the way that they work to continue delivery of services and take on new roles and responsibilities. Examples included using telemedicine as an alternative healthcare option, changing the way drugs were provided, and changing service delivery locations. These adaptations demonstrated high levels of personal commitment, including long working hours, as seen in other studies [17,21,22].

However, some adaptations came at personal cost for CHWs, including for their time and finances as well as mental health, suggesting that these adaptations are better viewed as ways of coping within a context of inadequate support, rather than positive resilience strategies. This signals the importance of ensuring health systems are robust, and so able to adapt without increasing population or health worker vulnerabilities [101]. Resilience should not be built on the back of a cadre that lacks system-driven support, rights and entitlements.

The article brings new evidence to the discussion on CHWs and COVID-19 and covers varied country contexts. The paper does not aim to represent the experiences of all CHW or of CHW in all countries. However, similarity of some finding with those in other articles on CHW and COVID-19 suggests some CHW experiences reported in this article are likely to be present in other contexts. However, our analysis has limitations. The nature of the pandemic and country response have changed over time, affecting CHWs’ roles, challenges and adaptations; the last report used in this synthesis was completed in November 2021, and CHWs experience may have since changed. In addition, each study had a specific purpose, and CHWs’ roles during COVID-19 were more central to some studies than others. As such, the reports do not provide directly comparable information across countries, and varied focus and purpose of the original reports means the depth and level of detail on CHWs experiences varied. Lack of evidence on a particular aspect in one country may therefore mean a gap in information in the reports, rather than indicating that this experience was not shared by CHWs in that context.

One strength of the paper is that it makes available evidence from consultancy reports that are often not accessible; few of the reports used for this synthesis were in the public domain, and none were published in the academic literature. However, the synthesis is restricted to information from research where OPM was involved, and additional evidence could be provided through a wider set of material and full evidence review. We hope this paper can contribute material for future, wider evidence synthesis by the global health community.

Finally, the heterogeneity in CHWs across cadres and countries (for example, in employment status, training and responsibilities as well as personal or household characteristics means significant diversity in CHWs experiences during COVID-19. The effects of this diversity could not be fully examined in this synthesis, but are an important avenue for further intersectional analysis.

## Conclusion

CHW are critical for effective response to disease outbreaks, including pandemics like COVID-19. Their interface position between the community and the health system enables them to provide services to the most marginalised groups. However, this is not without challenges. To support CHWs’ contribution in providing essential services during an emergency and to protect their wellbeing, CHWs need adequate resources, managerial and financial support, and recognition of their role and challenges at all stages of the policy cycle.

## Supporting information

S1 Table Reports included in the synthesis. (PDF)

S2 Table Thematic framework for synthesis. (PDF)
