# Supplementary material for: Community health workers and Covid-19: Cross-country evidence on their roles, experiences, challenges and adaptive strategies
Source: PLOS Glob Public Health. 2023 Jan 4;3(1):e0001447. doi: 10.1371/journal.pgph.0001447 (PMC10022071; doi:10.1371/journal.pgph.0001447)
Supplement: S1 Table — (PDF) [file pgph.0001447.s001.pdf]

S1 Table. Reports included in the synthesis

Project	Funder and client	Title	Country	Month/Year	Focus	Methods	Authors	Key organisational partners (for reports where other organisations beyond OPM were involved in co-producing the research)	Ethical approval
Assessing the Indirect Effects of COVID-19 on Essential Health and Nutrition Services in selected rural and urban settings of Bangladesh	Funded by the Global Financing Facility, managed by the World Bank	Assessing the indirect effects of COVID-19 on Essential Health and Nutrition Services in selected rural and urban settings of Bangladesh: A qualitative study	Bangladesh	May 2021	Qualitative assessment of the indirect effects of COVID-19 on Essential Health and Nutrition Services (EHNS) in selected rural and urban settings with specific focus on RMNCAH provision, coverage, utilisation and care-seeking.	Document review; 68 in-depth interviews with health service providers (including HA, FWA, FWV, other health workers, and managers, in the government, private, NGO and informal sectors); 4 in-depth interviews with community leaders; focus group discussions with CHW (HAs (1), FWAs (1), FWVs (1), BRAC	Muhammod Abdus Sabur, Nazme Sabina, and Adiba Khaled	Development Research Initiatives (dRi)	Approval from the Institutional Review Board of the Institute of Health Economics, University of Dhaka, which is approved by Federal Wide Assurance (FWA), [No. FWA00026031].

Project	Funder and client	Title	Country	Month/Year	Focus	Methods	Authors	**Key organisational partners** (for reports where other organisations beyond OPM were involved in co-producing the research)	Ethical approval
						shebikas (2) (DOTS providers), and community members and patients (34); 25 key informant interviews with national government, development agencies, and other key stakeholders and experts.			
Maintaining essential services after a natural disaster	Foreign, Commonwealth & Development Office (FCDO)	COVID-19 Rapid country studies: country reports for Bangladesh, Kenya, and Sierra Leone, cross-country synthesis report covering these countries, Pakistan and Uganda	Bangladesh, Kenya, Pakistan, Sierra Leone, Uganda	July 2020	Rapid situation analyses on the initial response to COVID-19 in the first few months of the outbreak, considering different health system pillars, wider governance structures, and response in related sectors	Document review, cross-country total of 52 key informant interviews with policy makers and other stakeholders; community survey in Sierra Leone	Debbie Hillier, Tom Newton-Lewis, Rithika Nair, and Christoph Larsen		The rapid COVID-19 assessments were short, initial assessments conducted to inform immediate programming decisions. The methods were restricted to document review and discussions with senior officials on health system issues, within their official professional roles,

Project	Funder and client	Title	Country	Month/Year	Focus	Methods	Authors	Key organisational partners (for reports where other organisations beyond OPM were involved in co-producing the research)	Ethical approval
					such as social protection				which did not cover sensitive or personal information. Given this purpose and methods, ethics review was not required.
		COVID-19 Response: Rapid country study Bangladesh	Bangladesh	April 2020	Rapid assessment on leadership and governance of the response to COVID-19 and impact of COVID-19 across social sectors, with a primary focus on health	Document review, and 5 key stakeholder interviews	Shah Monir Hossain and Rumana Huque		As above for the Maintains COVID-19 Rapid country studies
		Response & Preparedness for Essential Health and Nutrition Services During Disasters In Pakistan	Pakistan	June 2021	Health system preparedness and response to shocks (floods droughts COVID-19), considering different health system pillars at national and province level, and community response	Document review; review of HMIS data; key informant interviews with government and other stakeholders involved in health sector response to shocks at national (14), provincial (81)	Zafar Fatmi, Rabia Najmi, Nousheen Akber Pradhan, Zarak Ahmed, Minhaj Qidwai, Sana Hyat, and Sania Khursheed	Department of Community Health Sciences Aga Khan University, Karachi (Pakistan)	Approval from the Ethical Review Committee of the Aga Khan University [2020-1936-10170] and ethical clearance from the National Bioethics Committee. [Ref: No.4-87/COVID-29/ NBC/21/]

Project	Funder and client	Title	Country	Month/Year	Focus	Methods	Authors	**Key organisational partners** (for reports where other organisations beyond OPM were involved in co-producing the research)	Ethical approval
						district (63) and village or union (48) levels; structured interviews with 63 healthcare providers; focus group discussions with Lady Health Workers (LHWs) (16) and male and female community members (38)			
		Rapid Country Study: Sierra Leone	Sierra Leone	May 2020	Rapid assessment on leadership and governance of the response to COVID-19 and impact of COVID-19 across social sectors, with a primary focus on health	Document review; 12 key informant interviews with national government, donors, NGOs and youth leaders; observation of EOC meetings.	Kevin Grieco, Yasmina Yusuf, and Niccoló Meriggi	Individual consultants	As above for the Maintains COVID-19 Rapid country studies

Project	Funder and client	Title	Country	Month/Year	Focus	Methods	Authors	**Key organisational partners** (for reports where other organisations beyond OPM were involved in co-producing the research)	Ethical approval
		The effectiveness of the Sierra Leone health sector response to health shocks: Evidence from the COVID-19 perception survey	Sierra Leone	May 2021	The health sector response to COVID-19, particularly related to service delivery, leadership and governance, the health workforce, and community ownership and participation.	Survey of 303 stakeholders engaged with the COVID-19 response, including Ministry of Health and Sanitation employees at national, district and facility levels, and staff from other government or partner agencies.	Philip S. Amara, Fredline A M'Cormack-Hale, Mohamed Kanu, Regina Bash-Taqi, and Alhassan Kanu	Institute for Development, International Growth Centre, Dalan Development Consultants and College of Medicine and Allied Health Sciences	Approved by the Sierra Leone Ethics and Scientific Review Committee (no reference numbers used)
		Beyond the state: The role of traditional leaders in COVID-19	Sierra Leone	September 2020	Role of traditional leaders in supporting the COVID-19 response at community level, including extent of and gaps in government coordination	Key informant interviews with 15 district-level government actors, traditional leaders, and health professionals	Kevin Grieco	International Growth Centre, Dalan Development Consultants and College of Medicine and Allied Health Sciences	Approved by the Sierra Leone Ethics and Scientific Review Committee (no reference numbers used)

Project	Funder and client	Title	Country	Month/Year	Focus	Methods	Authors	**Key organisational partners** (for reports where other organisations beyond OPM were involved in co-producing the research)	Ethical approval
		COVID-19 Response: Rapid country study Kenya	Kenya	May 2020	To learn from COVID-19 around the national ability to respond to shocks. by focusing on the leadership and governance of the response to the pandemic, and its impact across social sectors, with a primary focus on the health sector.	Documents review; 6 remote interviews with key stakeholders.	Akaco Ekirapa		As above for the Maintains COVID-19 Rapid country studies
The Bihar Technical Support Programme - learning grant	Bill and Melinda Gates Foundation (BMGF)	Bihar COVID-19: Experiences of Community Women (multiple reports)	India	June 2020-December 2020	Gender-based impacts of COVID-19 on the health, social lives, livelihoods and other aspects of women's lives during COVID-19	Series of telephone interviews (5 rounds) with female self-help group leaders, members and community mobilisers, ranging from 8-12 women per round.	Priya Das, Rochana Kammowanee, and Maheen Shakeel		Approved by the Sigma Institutional Review Board: [IRB Number: 10011/IRB/20-21].

Project	Funder and client	Title	Country	Month/Year	Focus	Methods	Authors	**Key organisational partners** (for reports where other organisations beyond OPM were involved in co-producing the research)	Ethical approval
		Bihar COVID-19 Situation: PHC Preparedness and Impact on Services (multiple reports).	India	April 2020 – May 2020	Barriers and facilitators to the primary care response to COVID-19, the ability of rural health to provide routine essential services, and the vulnerabilities faced by health providers.	Document review; series of informal telephone conversations with Community Health Centre managers and frontline workers, including ANMs	Priya Das, Karima Khalil, and Priyanjali Mitra		Approved by the Sigma Institutional Review Board [IRB Number: 10026/IRB/19-20]
	Bill and Melinda Gates Foundation (BMGF)	Understanding the Community Health System: Experiences of Elected Women Representatives (EWRs).	India	December 2020	The role of local government Elected Women Representatives (ERWs) in the community health system, including their links with CHW, including their role during COVID-19	Telephone interviews with 20 EWRs	Vinaya Padmanabhan, Shuchi Srinivasan, Bibha Mishra. and Rajiv Bhardwaj, Bhagwati Pandey	Centre for Catalysing Change (C3)	Approved by the Sigma Institutional Review Board [IRB Number: 10027/IRB/20-21].

Project	Funder and client	Title	Country	Month/Year	Focus	Methods	Authors	**Key organisational partners** (for reports where other organisations beyond OPM were involved in co-producing the research)	Ethical approval
		Making community health worker supervision more supportive: insights from an implementation research pilot.	India	November 2021	The impact of training in supportive supervision alongside support for coaching and mentoring on the perceptions and performance of CHWs and their supervisors.	Telephonic interviews with 3 BCMs, 6 AFs and 12 ASHAs; in-person quantitative survey with 708 ASHAs and 34 Afs.	Shuchi Srinivasan, Arpana Kullu, Bhagwati Pandey, Adiba Khaled, Agrima Sahore, and Cindy Carlson		Approved by the Sigma Institutional Review Board [IRB Number: 10012/IRB/21-22].
Assessment of Institutional Arrangements and Human Resources of the National Tuberculosis Elimination Program	World Bank	Assessment of Institutional Arrangements and Human Resources of the National Tuberculosis Elimination Program (NTEP).	India	February 2021	Institutional support mechanisms to implement the NTEP at national, state and district levels, with a focus on structures and gaps related to human resources, and including the impact of COVID-19 on TB services	Document review; quantitative telephone survey of 377 health managers and providers involved in core TB or general health service delivery, from state to primary level (including ASHA), to assess workload using the	**OPM**<br>Vimal Kumar, Agrima Sahore, Soumyajit Ray, Adiba Khalid<br><br>**OPM External**<br>Shankar Dapkerkar, Shveta Kalyanwala<br><br>**KIT**<br>Marjolien Dielemen, Christina Mergenthaler	Ministry of Health and Family Welfare (MOHFW)-GoI, and National Health Systems Resource Centre (NHSRC), Royal Tropical Institute (KIT), National Tuberculosis Elimination Program (NTEP).	Approved by the Sigma Institutional Review Board [IRB Number: 10032/IRB/20-21]

Project	Funder and client	Title	Country	Month/Year	Focus	Methods	Authors	**Key organisational partners** (for reports where other organisations beyond OPM were involved in co-producing the research)	Ethical approval
						WHO Indicator of Staffing Need; 60 qualitative telephone interviews with health managers and providers from state to block level, on experiences and challenges in TB service provision and staffing, and additional discussions with national government and donors			
Building Resilience in Ethiopia – Technical Assistance	Foreign, Commonwealth & Development Office (FCDO) and U.S. Agency for International Development (USAID)	Intra-Action Review (IAR) on Public Health Preparedness and Response to Covid-19 in Ethiopia	Ethiopia	October 2020	Effective practice and challenges in the COVID-19 response, to guide future action for the response and future preparedness	Document review; key informant interviews, focus group discussions and workshops with government staff involved	Ministry of Health – Ethiopia<br><br>Ethiopian Public Health Institute	Ethiopia Ministry of Health, Ethiopian Public Health Institute, Regional Health Bureaus	Intra-Action Reviews are led by government and conducted as per WHO's Guidance for Conducting a Country COVID-19 Intra-Action Review. They involve discussion among key COVID-19 responders and decision makers

Project	Funder and client	Title	Country	Month/Year	Focus	Methods	Authors	**Key organisational partners** (for reports where other organisations beyond OPM were involved in co-producing the research)	Ethical approval
						in the response at national, regional, and facility level			within government, and focus on systems improvements rather than any sensitive or personal issues. Hence, ethics approval is not recommended or required for IARs.
	Foreign, Commonwealth & Development Office (FCDO) and U.S. Agency for International Development (USAID)	Intra-Action Review (IAR) on Public Health Preparedness and Response to Covid-19 in Sidama, Ethiopia	Ethiopia	March 2021	Effective practice and challenges in the COVID-19 response, to guide future action for the response and future preparedness	Document review; key informant interviews with government staff involved in the response at regional, or quarantine facility levels; small group workshops involving over 32 experts) from the regional public health emergency operations centre.	Sidama Regional Health Bureau,<br>Ministry of Health – Ethiopia<br>Ethiopian Public Health Institute	Sidama Regional Health Bureau, Ministry of Health, Ethiopian Public Health Institute	As above for national IAR

Project	Funder and client	Title	Country	Month/Year	Focus	Methods	Authors	**Key organisational partners** (for reports where other organisations beyond OPM were involved in co-producing the research)	Ethical approval
	Foreign, Commonwealth & Development Office (FCDO) and U.S. Agency for International Development (USAID)	Intra-Action Review (IAR) on Public Health Preparedness and Response to Covid-19 in Ethiopia. Gambella Region.	Ethiopia	March 2021	Effective practice and challenges in the COVID-19 response, to guide future action for the response and future preparedness	Document review; key informant interviews with public health emergency operations centre staff; focus group discussions involving 66 stakeholders, including staff from the regional public health emergency operations centre and other partners	Gambella Region Health Bureau, COVID-19 Public Health Emergency Operation Centre, WHO and Partners	Gambella regional Health Bureau, WHO	As above for national IAR
Operational Research on Key Nutrition-Specific Intervention in Ethiopia	United Nations Children's Fund (UNICEF)	Formative research on key nutrition specific interventions in Ethiopia. UNICEF Operational research on key nutrition specific interventions	Ethiopia	August 2021	Implementation of three nutrition-specific interventions, including gap and challenges, and the effects of COVID-19 on delivery	197 in-depth interviews with health professionals at regional, zonal, health centre and health post levels, including HEW, and community	Daniel Wate, Sonya Rabeneck, Alula Meressa Teklu, Girmay Medhin, Sarone Makonnen, Gloria Olisenekwu and Madhav Vaidyanathan.	Monitoring Evaluation, Research and Quality improvement consultancy - MERQ PLC (Ethiopia)	Approved by the Ethiopian Public Health Institute (EPHI) Institutional Review Board. [Protocol Number: EPHI-IRB-333-2020; EPHI reference number 6.13/285]

Project	Funder and client	Title	Country	Month/Year	Focus	Methods	Authors	**Key organisational partners** (for reports where other organisations beyond OPM were involved in co-producing the research)	Ethical approval
						members; 28 focus group discussion with female community members; observation at 56 facilities			
COVID-19 vaccine supply and rollout	United Nations Children's Fund (UNICEF)	Real time assessment (RTA) of UNICEF's ongoing response to COVID-19 in eastern and southern Africa: COVID-19 vaccine supply and rollout. November 2021	Ethiopia	December 2021	Assessment of UNICEF's support to COVID-19 vaccine rollout, including understanding progress and challenges in supply, distribution, and coordination	Document review; key informant interviews with 30 respondents, including staff from UNICEF, development partners and government at national and subnational levels, and regional UNICEF staff	Kate Gooding, Jayne Webster, Nicola Wiafe and Vimal Kumar		Approved by the Human Subjects Research and Ethics Approval Independent Review Board [IRB 2021: 389GLOB29x].
