# Supplementary material for: Community health workers and Covid-19: Cross-country evidence on their roles, experiences, challenges and adaptive strategies
Source: PLOS Glob Public Health. 2023 Jan 4;3(1):e0001447. doi: 10.1371/journal.pgph.0001447 (PMC10022071; doi:10.1371/journal.pgph.0001447)
Supplement: S2 Table — (PDF) [file pgph.0001447.s002.pdf]

S2 Table. Thematic framework for synthesis

Theme	Sub-theme
**1 Document Information**	1.1 Country
	1.2 Research Title
	1.3 Research Objectives
	1.4 Method
	1.5 Sample (numbers, types of stakeholders, locations including whether urban or rural
	1.6 Dates of fieldwork/data collection
	1.7 OTHER document details
**2 CHW cadre characteristics**	2.1 CHW cadre
	2.2 Typical Years of training for each cadre
	2.3 Mode of engagement for each cadre
	2.4 Payment mode for each cadre
	2.5 OTHER type of Support provided to CHW beyond payment
**3 Routine services provided by these CHW cadres (pre-covid) – specify for each cadre discussed in the report**	3.1 Preventive services
	3.2 Provision of clinical services
	3.3 Community mobilization and education
**4 Roles CHW played in the COVID-19 response, including vaccine delivery**	4.1 Identification of potential covid cases / screening people with potential COVID-19 symptoms
	4.2 Contact tracing
	4.3 Follow-up of patients with COVID-19
	4.4 Epidemographic surveillance and record-keeping
	4.5 Delivery of Covid Vaccines
	4.6 Community education and awareness on COVID-19
	4.7 OTHER Roles
**5 Support provided to CHW during COVID-19– including information on lack of this support**	5.1 Training on providing services related to Covid-19
	5.2 Training on providing routine services during COVID-19
	5.3 Guidelines to support CHWs activities related to Covid-19 or delivery of routine services during COVID-19
	5.4 Additional human resource

Theme	Sub-theme
	5.5 Financial motivation / support for CHWs
	5.6 Personal protective equipment (PPE)
	5.7 Managerial support for CHW during COVID-19
	5.8 Community support
	5.9 Peer Support
	5.10 Support for CHW mental health/ wellbeing
	5.11 Other types of support provided
	5.12 Other support requested or suggested by CHW
	5.13 Sources of information for CHW
	5.14 Technological Support
**6 Beyond the areas of support provided – or not provided - what other issues helped or hindered CHW experience in providing services during COVID-19 – including routine services and COVID-19-specific services?**	**System Level**
	6.1 Human resource
	6.2 Financial resources
	6.3 Essential medicines / routine vaccines
	6.4 Information systems
	6.5 Power Structures
	**Community Level**
	6.6 Community attitudes
	6.7 Community ability to access services
	**Family/Personal level**
	6.8 Domestic responsibilities and family support
	6.9 Financial circumstances
	**Wider issues beyond the health system**
	6.10 Movement restrictions
	6.11 Transportation
	6.12 Other enablers/barriers
	6.13 Enablers and Barriers in providing services during Covid-19
	6.14 Gendered hierarchies
**7 Wider system issues that affected support provided to CHW and their ability to conduct activities related to covid and to continue routine services**	7.1 Previous epidemic experience
	7.2 Support from development partners and level of coordination
	7.3 Government coordination and decision-making systems
	7.4 OTHER factors

Theme	Sub-theme
**8 Effectiveness of service delivery – COVID-19-specific services**	8.1 Effective delivery of COVID-19-related services by CHW
	8.2 Effectiveness Covid-19 Services- patient/community views
**9 Effectiveness of service delivery – routine CHW services**	9.1 Delivery of routine services during COVID-19 - changes in coverage
	9.2 Delivery of routine services during COVID-19 – changes in equitable coverage
	9.3 Delivery of routine services during COVID-19 - changes in quality
	9.4 Cease providing essential healthcare services
**10 CHW well-being during COVID-19**	10.1 Mental well-being of CHWs during COVID-19
	10.2 Effects on CHW workload
	10.3 Other effects on CHW wellbeing
**11 What adaptations enabled continued service delivery?**	11.1 Telemedicine
	11.2 Change in drug provision
	11.3 Change service location
	11.4 OTHER adaptations
